# Loop Closure with 3D Gaussian Splatting for Dynamic SLAM

**DOI:** 10.3390/s26092669

**Published:** 2026-04-25

**Authors:** Zhanwu Ma, Wansheng Cheng, Song Fan

**Affiliations:** 1 School of Electronic and Information Engineering, University of Science and Technology Liaoning, Anshan 114051, China; 2 Key Laboratory of Electric Drive and Control of Anhui Province, Wuhu 241000, China

**Keywords:** 3D gaussian splats, dynamic environments, loop closure, visual simultaneous localization and mapping

## Abstract

**Highlights:**

**What are the main findings?**

- The proposed LCD-Splat method integrates dynamic object detection, 3D Gaussian Splatting mapping, and a novel loop closure mechanism, significantly outperforming existing SLAM algorithms in tracking and reconstruction within dynamic environments.
- The introduction of an improved multi-view geometry constraint and a coarse-to-fine 3DGS registration algorithm effectively mitigates dynamic interference and enables accurate global map consistency.

**What are the implications of the main findings?**

- This work provides a robust, high-fidelity SLAM framework that advances 3D scene understanding and long-term autonomy for intelligent autonomous unmanned systems (iAUS) operating in complex, dynamic real-world settings.
- The methodology offers new insights for high-precision perception and mapping, directly contributing to the core technologies of navigation, guidance, and environmental awareness in unmanned systems.

**Abstract:**

Robust pose estimation and high-fidelity scene reconstruction in dynamic environments represent core challenges in the field of Visual Simultaneous Localization and Mapping (SLAM). Although 3D Gaussian Splatting (3DGS)-based techniques have demonstrated significant potential, existing methods typically assume static scenes and struggle to address the inconsistency between photometric and geometric observations in dynamic settings, leading to a notable degradation in pose estimation and map accuracy. To address these issues, this paper presents a novel dynamic SLAM method: Loop Closure with 3D Gaussian Splatting for Dynamic SLAM (LCD-Splat). Taking RGB-D images as input, LCD-Splat integrates Mask R-CNN with an improved multi-view geometry approach to detect dynamic objects, generating static scene maps and filling in occluded backgrounds. By leveraging 3DGS submaps and a frame to model tracking strategy, LCD-Splat achieves dense map construction. The method initiates online loop closure detection and employs a novel coarse to fine 3DGS registration algorithm to compute loop closure constraints between submaps. Global consistency is ultimately ensured through robust pose graph optimization. Experimental results on real-world datasets such as TUM RGB-D and Bonn demonstrate that LCD-Splat outperforms existing state-of-the-art SLAM methods in terms of tracking, scene reconstruction, and rendering performance. This approach provides novel insights for high-precision SLAM in dynamic environments and holds significant implications for scene understanding in complex settings.

## 1. Introduction

Visual SLAM is a key technology that enables robots to perform 3D map reconstruction and camera pose estimation in unknown environments [1]. It is widely applied in fields such as autonomous navigation, autonomous driving, virtual reality, and augmented reality [2]. However, SLAM systems in real-world applications must address the complex challenges in dynamic environments [3], including semantic segmentation of dynamic objects, loop closure detection, and trajectory drift correction [4]. These challenges impose higher demands on SLAM’s rendering performance and trajectory estimation accuracy. Despite significant advancements in SLAM technology for static environments [5], most research still relies on the assumption of static environments, lacking loop closure detection or Global Bundle Adjustment (BA), which leads to poor global consistency of the scene [6]. Especially in dynamic environments, achieving robust pose estimation, high-quality map reconstruction, and global consistency optimization remains a critical unsolved challenge [7].

In recent years, Neural Radiance Fields (NeRF) [8], as an implicit representation technique, has been introduced to the SLAM field, demonstrating great potential with its efficient dense scene modeling capability. For example, iMAP [9] was the first to utilize Multi-Layer Perceptron technology to apply NeRF for map representation in SLAM, achieving real-time scene modeling and tracking without prior knowledge. On the other hand, NICE-SLAM [10] achieves high-precision reconstruction of large-scale indoor scenes through hierarchical scene representation and geometric prior optimization. These methods significantly improve the density and continuity of SLAM systems, but their robustness in dynamic environments remains insufficient. Moreover, these approaches typically require predefined scene boundaries to initialize the neural voxel grid, and implicit representations still face challenges in information fusion and editing.

To overcome these limitations, explicit representation techniques have gradually been introduced into SLAM systems, with 3D Gaussian splatting [11] becoming a research focus due to its continuous, smooth, and differentiable geometric representation. GS-SLAM [12] was the first to apply 3D Gaussian representation to SLAM. Through a real-time differentiable splatting rendering pipeline, it accelerates map optimization and RGB-D rendering, efficiently reconstructing new scene geometry and improving the mapping of previously explored areas. Subsequently, SplaTAM [13] achieved high-fidelity scene reconstruction using explicit volumetric representation and supports RGB-D camera input without pose information. Despite significant advancements in SLAM systems for static environments, a notable research gap remains in dynamic scenarios. While recent 3D Gaussian Splatting-based SLAM approaches have improved reconstruction quality, they predominantly rely on the assumption of static scenes and lack robust loop closure mechanisms designed for dynamic environments, resulting in accumulated drift and degradation of global consistency. Moreover, the integration of 3DGS representation with online loop detection and constraint optimization represents a critical yet underexplored gap in current SLAM research. To address these issues, this paper proposes a novel 3D Gaussian splatting-based visual SLAM method, specifically designed for dynamic indoor scenes. The new method combines deep learning-based Mask R-CNN semantic segmentation, improved multi-view geometry detection, and 3D Gaussian splatting technology. The main contributions of this paper can be summarized as follows:

1. The innovative LCD-Splat is proposed, a 3D Gaussian Splatting SLAM for dynamic environments. This method integrates deep learning-based Mask R-CNN, improved multi-view geometry, 3D Gaussian Splatting registration, and loop closure detection modules, significantly enhancing camera tracking and 3D scene reconstruction accuracy.
2. Improved multi-view geometry. To address the issue that existing multi-view geometry constraints often overlook potential moving objects (such as balloons), an improved multi-view geometry constraint method is proposed.
3. A coarse-to-fine 3DGS registration algorithm is proposed, which accurately computes the relative loop closure constraints between submaps, thereby providing robust edge constraints for pose graph optimization.

## 2. Related Work

Dynamic Object-Aware Visual SLAM. Achieving robust SLAM in dynamic environments has always been a significant challenge in the field of visual SLAM. The classic method ORB-SLAM3 [14], based on Maximum A Posteriori estimation, provides real-time robust performance in a variety of indoor and outdoor environments. However, the performance of ORB-SLAM3 in dynamic scenes is limited, as it cannot effectively handle the interference from dynamic objects. This limitation has driven the development of SLAM methods specifically designed for dynamic environments. To improve localization and mapping accuracy, DynaSLAM [15] further combines deep learning with multi-view geometry to detect and remove dynamic objects, allowing the SLAM system to focus more accurately on mapping the static parts of the scene. Subsequently, EM-fusion [16] proposed a dynamic SLAM method based on dense object-level SDF representation. By using probabilistic modeling, it directly aligns RGB-D images with SDF, effectively addressing data association and occlusion handling issues in multi-object tracking. To improve the detection capability of dynamic elements, Refusion [17] introduced a fusion method that combines residual information after initial registration with explicit free-space modeling. This not only significantly enhances the mapping performance in dynamic scenes but also enables efficient detection of dynamic elements. Meanwhile, MID-Fusion [18] adopted a multi-instance segmentation method using object-level octree volumetric representation, combining geometric and motion information to optimize the boundaries of segmentation masks, thereby further improving the accuracy of dynamic object modeling. In distinguishing dynamic from static points, LC-SLAM [19] proposed a graph-cut RANSAC-based method, which enhances the detection accuracy of dynamic 3D feature points by utilizing unary potential priors in conditional random fields. Recently, YOLO-ORB-SLAM3 [20] integrated the YOLOv5 object detection module into the ORB-SLAM3 framework, enabling better detection and handling of dynamic objects in dynamic environments.

Implicit SLAM Using Neural Radiance Fields. In recent years, with the widespread attention given to neural implicit scene representations due to their exceptional expressive power and efficient memory utilization, the RGB-D SLAM field has witnessed a new wave of research. Various SLAM methods based on neural implicit representations have been proposed, enhancing the performance of dense visual SLAM through different designs. In contrast, ESLAM [21] further integrates Neural Radiance Fields (NeRF) into the SLAM system, processing RGB-D frames with unknown camera poses in a sequential manner, thereby achieving an efficient and precise dense visual SLAM method. To further innovate, GO-SLAM [22], based on a deep learning framework, supports frame-by-frame optimization with efficient loop closure detection and online global bundle adjustment. Addressing the challenges in dynamic environments, RoDyn-SLAM [23] proposes a motion mask generation method that combines optical flow and semantic masks, effectively filtering out invalid sampled rays, thereby significantly improving the accuracy of masks in dynamic scenes.

SLAM with 3D Gaussian Splatting Representation. In recent years, 3D Gaussian Splatting has demonstrated tremendous potential in the field of 3D vision due to its efficient rasterization processing and flexible editing capabilities based on explicit representations. It has gradually emerged as a promising technology to replace NeRF. Building on this technology, several studies have further advanced its application and development in SLAM systems. First, Gaussian-SLAM [24] proposed an innovative method of organizing the scene into submaps, achieving precise camera tracking from frames to models by minimizing the photometric and geometric loss between input frames and rendered frames. Furthermore, Gaussian Splatting SLAM [25], based on Gaussian explicit representations, integrates geometric validation and regularization mechanisms to effectively address the ambiguity issues in incremental 3D dense reconstruction. In addition, LoopSplat [26] introduced the first SLAM framework capable of triggering loop closure online. This method directly computes the relative loop closure constraints between submaps through 3D Gaussian Splatting registration. Recently, GS-ICP-SLAM [27] proposed a dense SLAM method that combines Generalized Iterative Closest Point (G-ICP) with 3D Gaussian Splatting. This method not only enhances robustness in static scenes but also optimizes the reconstruction quality of dense maps, providing reliable technical support for indoor navigation and industrial robot applications.

It is worth noting that within the broader landscape of robust localization research, existing studies have addressed the challenges of dynamic scenarios from different perspectives. For example, Ref. [28] proposed a VINS enhancement method based on intelligent feature grading, which balances dynamic feature rejection and static information preservation through adaptive evaluation of feature quality; Ref. [29] improved localization robustness in complex urban environments for autonomous driving systems via adaptive VINS-GNSS fusion and failure detection mechanisms. These methods primarily handle dynamic interference at the feature level or sensor fusion level, whereas our work focuses on achieving dynamic robustness at the scene representation level. Specifically, instead of directly manipulating traditional feature points, LCD-Splat operates on the 3D Gaussian representation, suppressing dynamic Gaussians through multi-view geometric consistency checks and dynamic probability propagation while preserving complete geometric and appearance information of the scene. Moreover, the proposed loop closure optimization mechanism enhances long-term system consistency through submap registration in dynamic environments, even without external absolute observations (e.g., GNSS). Therefore, our method provides a technical pathway complementary to feature-level and sensor-level approaches at the representation and optimization level of dynamic SLAM, offering a novel solution for robust localization in dynamic settings.

## 3. System Description

**Figure 1:**
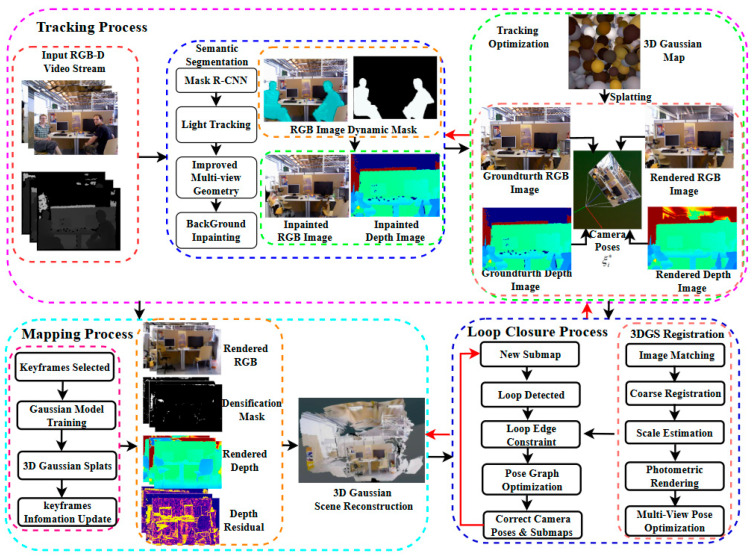
System Architecture. Dynamic RGB-D frames are used to generate a static 3D Gaussian map through semantic segmentation and loop closure detection while optimizing the camera pose, represented by Lie algebra $\xi_{i}$.

The system architecture, as shown in Figure 1, consists of three main modules: Tracking, Mapping, and Loop Closure. First, the Tracking Process receives the RGB-D video stream from the visual sensor. Through Mask R-CNN and an improved multi-view geometry technique, it performs precise semantic segmentation of dynamic objects in the images. The segmentation results generate an RGB-D dynamic mask, and the background inpainting technique is used to restore the masked regions of the RGB and depth images. Subsequently, the system uses a frame-to-model tracking method to accurately localize the current frame within the submap. By minimizing the differences in RGB and depth images between the current frame and the rendered frame, it performs camera pose estimation. When a new submap is generated, the Loop Closure Process is triggered. In this process, a coarse-to-fine 3DGS registration algorithm is used to provide accurate loop edge constraints for loop closure detection. Subsequently, robust pose graph optimization is applied to correct the camera pose and submap positions, ensuring global consistency. Finally, the Mapping Process selects appropriate keyframes and performs efficient Gaussian model training, using 3D Gaussian Splatting technology for precise 3D scene reconstruction.

## 4. Method

LCD-Splat is an RGB-D SLAM system that constructs a globally consistent 3D Gaussian map from RGB-D input frames while simultaneously estimating camera poses. This section first reviews the 3D Gaussian Splatting SLAM system, which serves as the core foundation of LCD-Splat. It then provides a detailed explanation of the semantic segmentation module for dynamic objects, including Mask R-CNN and the improved multi-view geometry algorithm. Next, the algorithmic process of the proposed coarse-to-fine 3DGS registration module is derived. Finally, it describes how Loop Closure Detection is achieved through this registration module and seamlessly integrated into the Gaussian-SLAM system.

### 4.1. 3D Gaussian Splatting SLAM

#### 4.1.1. 3D Gaussian Representation

3D Gaussian Splatting is an efficient method for 3D scene reconstruction. It utilizes video streams captured from different viewpoints and iteratively optimizes the 3D Gaussian model parameters using differentiable characteristics. The 3D scene is represented by a set of Gaussian ellipsoids.

To generate depth and color renderings, the 3D Gaussian ellipsoid ($\mu_{w},\Sigma_{w}$) in the world coordinate system is projected onto the 2D image plane, resulting in a 2D Gaussian distribution ($\mu_{I},\Sigma_{I}$). The Gaussian Splatting mean $\mu_{I}$ is calculated as follows (1) [30]:

(1) $$\mu_{I}=\pi \left(P\cdot T_{w}^{c}\cdot \mu_{w}\right)$$

where π represents the pixel coordinate projection function of ${\mathbb{R}}^{4}\to {\mathbb{R}}^{2}$; $P\in {\mathbb{R}}^{4\times 4}$ represents the projection matrix; and $T_{w}^{c}\in SE\left(3\right)$ represents the transformation matrix from the world coordinate system to the camera coordinate system. The covariance $\Sigma_{w}$ of Gaussian Splatting is calculated as follows in Equation (2) [31]:

(2) $$\Sigma_{I}=J\cdot R_{w}^{c}\cdot \Sigma_{w}\cdot {R_{w}^{c}}^{T}\cdot J^{T}$$

where $J\in {\mathbb{R}}^{2\times 3}$ is the affine transformation matrix, approximated as the Jacobian matrix; and $R_{w}^{c}\in SO\left(3\right)$ is the rotational part of $T_{w}^{c}$.

#### 4.1.2. 3D Gaussian Submap

To save memory overhead, following the approach in Ref. [24], the 3D Gaussian scene is stored as a series of submaps. Each submap contains multiple keyframes observing a specific area and is represented by an independent 3D Gaussian point cloud. The Gaussian point cloud $P^{S}$ of each submap consists of n 3D Gaussians, expressed as follows in Equation (3):

(3) $$P^{S}=\left\{G_{i}\left(\mu ,\Sigma ,o,C\right)|i=1,2,3,\ldots ,n\right\}$$

This paper adopts the strategy from Ref. [30] for submap initialization, where a new submap is created if the translation of the current frame relative to the first frame of the active submap exceeds a predefined threshold t, or if the estimated Euler angles exceed the threshold θ. Based on the pose estimation of the current new keyframe, a dense 3D Gaussian point cloud is computed from the input RGB and depth images. The 3D Gaussian point cloud from the new keyframe is then added to the currently active submap to update the observed scene. Whenever a new 3D Gaussian point cloud is added to the active submap, all 3D Gaussian point clouds in the submap undergo a joint optimization of the total loss function $L_{t}$ for a fixed number of iterations. $L_{t}$ consists of three components: depth, color, and regularization terms, which are calculated as shown in Equation (4):

(4) $$L_{t}=\lambda_{dep}\cdot L_{dep}+\lambda_{col}\cdot L_{col}+\lambda_{reg}\cdot L_{reg}$$

where $\lambda_{dep}$, $\lambda_{col}$, and $\lambda_{reg}$ are non-negative values representing the loss weights for each corresponding term. $L_{dep}$ represents the depth loss, which is calculated as shown in Equation (5):

(5) $$L_{dep}={\left|\tilde{D}-D\right|}_{1}$$

where $\tilde{D}$ and D represent the rendered and real depth maps, respectively. $L_{col}$ represents the color loss, which is calculated as a weighted combination of the $L_{1}$ loss and SSIM loss, as shown in Equation (6) [26]:

(6) $$L_{col}=\left(1-\lambda \right)\cdot {\left|\tilde{I}-I\right|}_{1}+\lambda \cdot \left(1-SSIM\left(\tilde{I},I\right)\right)$$

where $\lambda \in \left[0,1\right]$, $\tilde{I}$, and I represent the rendered and real RGB images, respectively. $L_{reg}$ represents the regularization term, which effectively prevents excessive stretching of 3D Gaussian voxels in sparse areas, and is calculated as shown in Equation (7) [25]:

(7) $$L_{reg}=\sum_{n\in N}\frac{{\left|s_{n}-\hat{s_{n}}\right|}_{1}}{N}$$

where $s_{n}\in {\mathbb{R}}^{3}$ is the scale of the 3D Gaussian, $\hat{s_{n}}$ is the average scale of the submap, and N is the number of 3D Gaussian voxels in the submap.

### 4.2. Semantic Segmentation for Dynamic Objects

#### 4.2.1. Mask R-CNN

Mask R-CNN [32] is an advanced instance segmentation network that can be used for precise detection and segmentation of dynamic objects. In this study, Mask R-CNN is applied to perform pixel-level semantic segmentation on the input RGB images to identify potential dynamic objects. Mask R-CNN not only outputs pixel-level semantic labels but also provides instance labels, which enhances its adaptability in dynamic scenes. The core idea of Mask R-CNN is to add a branch for generating pixel-level segmentation masks to the Faster R-CNN object detection framework, enabling it to simultaneously perform object detection and instance segmentation. In this study, the TensorFlow implementation provided by Matterport is used, and experiments are conducted based on a pre-trained model from the MS COCO dataset. This network is capable of segmenting multiple potential dynamic object categories (such as people, vehicles, animals, etc.), ensuring comprehensive and accurate detection. If there is a need to expand the detection categories, the network can be fine-tuned using new training data to adapt to the specific requirements of the scene. The output of Mask R-CNN is a matrix  m×n×l, where  m×n represents the resolution of the input image, and l denotes the number of detected object categories. For each category, the network generates a binary mask, and by combining all the masks, the pixel-level segmentation results of dynamic objects in the entire scene are obtained. This segmentation information can be used to further optimize the VSLAM system, enhancing its robustness and accuracy in dynamic environments.

After processing with Mask R-CNN, dynamic objects in the scene are segmented. However, some objects that are not initially classified as movable by the CNN-based detector—yet remain stationary for most observation periods—still need to be identified as potential dynamic instances. Accurate localization of such objects heavily relies on reliable camera pose estimation. To achieve this without introducing significant computational overhead, a low-cost camera tracking module has been designed. Specifically, this module adopts a simplified version of the frame-to-model tracking architecture illustrated in Figure 1. By reducing the number of iterative optimizations and employing a lightweight feature representation, the module significantly reduces computational load while maintaining the tracking efficiency required for real-time operation.

In terms of initialization, the tracking module is bootstrapped using the camera pose estimated from the preceding global registration stage. The initial pose is further refined with a constant-velocity motion prior and updated frame by frame. Experiments demonstrate that on the dynamic TUM RGB-D dataset, the module achieves an average absolute trajectory error of 0.030 m, reflecting its practical accuracy. To balance efficiency and pose estimation quality, the module’s design clearly distinguishes between real-time tracking and global consistency optimization in terms of computational allocation. At the front end of pose estimation, the system uses sparse keypoint matching and incremental pose prediction, which ensures inter-frame tracking stability while significantly lowering per-frame computational demand. The quality of pose estimation is safeguarded by a multi-level geometric verification mechanism, including local motion smoothness constraints and projection consistency checks, thereby enhancing computational efficiency without compromising tracking accuracy.

#### 4.2.2. Improved Multi-View Geometry

**Figure 2:**
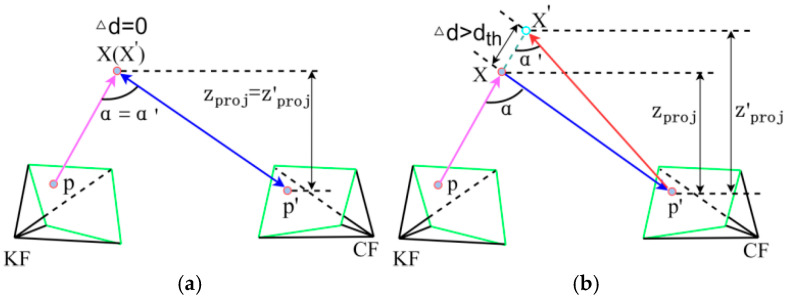
An improved multi-view geometry scheme. (**a**) The static point scenario; (**b**) The dynamic point scenario.

While Mask R-CNN can segment most dynamic objects, it is not used for tracking and mapping. However, this method may still miss some dynamic objects or fail to detect certain potential moving objects, as these objects are not predefined as dynamic but rather are movable—for example, rising balloons, moved boxes, carried books, and moving chairs, among others. The current dynamic detection module relies on a pre-trained semantic model. In the future, we will explore a strategy that integrates open-vocabulary segmentation with self-supervised motion learning to reduce dependency on fixed-category datasets and enhance adaptability to unknown dynamic objects. To address this issue, this paper proposes an Improved Multi-View Geometry, as shown in Figure 2, where (a) represents the static point scenario, and (b) represents the dynamic point scenario. For the current frame (CF), several keyframes (KF) with the highest overlap are selected. The selection of the number of overlapping keyframes directly impacts the accuracy and computational efficiency of dynamic point detection. Through experimental analysis, this study finds that an insufficient number of keyframes leads to inadequate geometric constraints, thereby increasing the risk of missed detection of dynamic points. Conversely, an excessive number of keyframes introduces noticeable computational redundancy, and as viewpoint differences expand and motion accumulation errors increase, the improvement in detection accuracy tends to saturate. When the number of keyframes is set to 5, the system achieves a favorable balance between detection performance and computational efficiency. Therefore, we ultimately determine 5 keyframes as the number of overlapping keyframes. This configuration demonstrates strong overall performance in subsequent experiments, effectively detecting dynamically defined objects without semantic labels while imposing no significant computational burden on the system.

Then, using the depth map and camera pose, the 3D coordinates X of each feature point p in the world coordinate system are calculated for the previous keyframe. The projection of these feature points onto the current frame is then computed to obtain the 3D coordinates $X^{\prime }$ of the feature point $p^{\prime }$ in the world coordinate system. The detailed calculation steps are as follows: First, the 3D coordinates X of the feature point p in the world coordinate system are calculated for the previous keyframe, as shown in Equation (8):

(8) $$X=T_{KF}\cdot \left(D_{p}\cdot \left(K^{-1}\cdot p_{\left(\mu ,\upsilon \right)}\right)\right)$$

Next, the projection of the 3D coordinates X onto the current frame is calculated, which corresponds to the pixel coordinates $p_{\left(\mu ,\upsilon \right)}^{\prime }$ of the feature point $p^{\prime }$, as shown in Equation (9):

(9) $$p_{\left(\mu ,\upsilon \right)}^{\prime }=K\cdot \left(T_{CF}\cdot X\right)$$

where $T_{CF}$ represents the camera pose transformation matrix of the current frame.

Finally, the 3D coordinates $X^{\prime }$ of the feature point $p^{\prime }$ in the world coordinate system are calculated for the current frame, as shown in Equation (10):

(10) $$X^{\prime }=T_{CF}\cdot \left(D_{p^{\prime }}\cdot \left(K^{-1}\cdot p_{\left(\mu ,\upsilon \right)}^{\prime }\right)\right)$$

where $D_{p^{\prime }}$ represents the depth information of the feature point $p^{\prime }$ in the depth map.

Using the 3D coordinates X and $X^{\prime }$ in the world coordinate system, the back-projection angles between feature points p and $p^{\prime }$ are calculated, specifically their disparity angles α and $\alpha^{\prime }$. If either disparity angle is greater than 30° or if the difference between the two disparity angles, denoted as $\Delta \alpha =\left|\alpha -\alpha^{\prime }\right|$, exceeds a predefined threshold θ, the feature point $p^{\prime }$ is determined to be part of a dynamic point. We conducted a statistical analysis of static frames from the TUM RGB-D and Bonn datasets. The results show that for static feature point pairs, over 95% of the absolute disparity angles are below 25°. Taking into account sensor noise and calibration errors, we set the disparity angle threshold to 30°as a conservative boundary for dynamic point discrimination. This threshold effectively covers the vast majority of static points. Furthermore, the threshold θ is determined based on statistical hypothesis testing: if Δα>θ, the two points are considered to have inconsistent motion patterns and are highly likely to be dynamic. By analyzing the distribution of Δα for static point pairs, we take the upper limit of the 95% confidence interval of this distribution as the predefined threshold θ, thereby achieving a better balance between false positives and missed detections. However, experiments showed that static objects were misidentified as dynamic objects due to viewpoint differences in the TUM RGB-D and Bonn datasets. Building upon this, the projections of the 3D coordinates X and $X^{\prime }$ along the *z*-axis, denoted as $z_{proj}$ and ${z^{\prime }}_{proj}$, are calculated. When the difference between the two projections, $\Delta z=\left|z_{proj}-{z^{\prime }}_{proj}\right|$, exceeds a predefined threshold τ, the feature point $p^{\prime }$ is classified as a dynamic point. To prevent the missed detection of movable objects, the Euclidean distance Δd between the 3D coordinates X and $X^{\prime }$ is calculated. If Δd exceeds a predefined threshold $d_{th}$, the feature point $p^{\prime }$ is classified as a dynamic point. Since depth error increases with distance, the threshold $d_{th}$ is set as a linear function of the depth D, as shown in Equation (11):

(11) $$d_{th}=d_{base}+\lambda \cdot D$$

where $d_{base}$ is the baseline value of the threshold, and λ is the linear growth coefficient related to depth D, used to adjust the threshold as it changes with depth.

**Figure 3:**
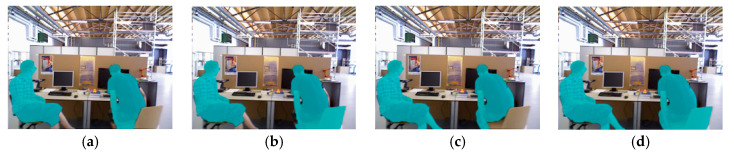
Dynamic object detection and segmentation in the TUM RGB-D dataset. (**a**) Traditional Multi-View Geometry. (**b**) Improved Multi-View Geometry. (**c**) Mask R-CNN. (**d**) Mask R-CNN and Improved Multi-View Geometry.

**Figure 4:**
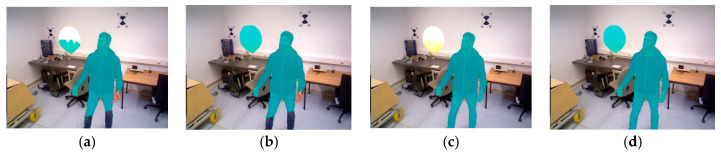
Dynamic object detection and segmentation in the Bonn dataset. (**a**) Traditional Multi-View Geometry. (**b**) Improved Multi-View Geometry. (**c**) Mask R-CNN. (**d**) Mask R-CNN and Improved Multi-View Geometry.

Some feature points located on the edges of moving objects may be incorrectly labeled as dynamic, which can cause issues. To address this, the information provided by the depth map is used. If a feature point is labeled as dynamic but its surrounding area exhibits a high variance in the depth map, its label is changed to static. At this point, the segmentation of dynamic and static objects is complete. Figure 3 and Figure 4 show examples of dynamic object masks applied to the TUM RGB-D and Bonn real-world datasets, respectively.

By analyzing Figure 3 and Figure 4, it can be observed that different methods exhibit varying performance in dynamic object detection. Using the (a) Traditional Multi-View Geometry method, the chair in Figure 3a on the right, which is being moved by a person, is not fully detected. In Figure 4a, only half of the rising balloon is detected. Additionally, the people in both images are not fully segmented, which may be related to the insufficient detection conditions for dynamic objects in this method. Using the (b) Improved Multi-View Geometry method, the chair being moved by a person in Figure 3b and the rising balloon in Figure 4b are both successfully detected. This is because, in this study, additional recognition conditions for dynamic objects were incorporated into the traditional method, thus improving the robustness of the detection. However, the method still fails to accurately segment dynamic people, which may be due to the sparse or indistinct feature points in these areas. Using the (c) Mask R-CNN method, the dynamic people in Figure 3c and Figure 4c are successfully detected. However, the chair being moved by a person and the rising balloon are not recognized, as these objects are not defined as dynamic targets in the prior knowledge, leading to them being incorrectly handled during the tracking and mapping process. Using the (d) Mask R-CNN and Improved Multi-View Geometry method, the dynamic people, the chair being moved by a person, and the rising balloon in Figure 3d and Figure 4d are all successfully detected. This indicates that the combination of the two methods effectively compensates for each other’s limitations, achieving comprehensive dynamic object detection and segmentation.

In the quantitative evaluation of the TUM RGB-D dynamic sequences, the proposed fusion strategy combining Mask R-CNN and the improved multi-view geometry achieves significant advantages. Compared to using only Mask R-CNN (Recall: 88.7%, Precision: 84.9%) or only the improved multi-view geometry (Recall: 77.2%, Precision: 90.1%), the fusion strategy improves both recall and precision, reaching 91.3% and 92.4%, respectively. Specifically, recall increases by 2.6 percentage points over Mask R-CNN alone, and precision improves by 2.3 percentage points over the improved multi-view geometry alone. These results indicate that the fusion method effectively reduces missed detections while suppressing false positives, significantly enhancing the overall robustness of dynamic detection.

In the quantitative evaluation of the Bonn dynamic sequences, the fusion strategy also demonstrates excellent generalization performance. Compared to using only Mask R-CNN (Recall: 87.1%, Precision: 81.5%) or only the improved multi-view geometry (Recall: 74.8%, Precision: 88.9%), the fusion strategy achieves 91.0% recall and 90.8% precision. Here, recall improves by 3.9 percentage points over Mask R-CNN alone, and precision increases by 1.9 percentage points over the improved multi-view geometry alone. This outcome further validates that the proposed method exhibits strong adaptability and stability across different dynamic scenarios.

#### 4.2.3. Background Inpainting

**Figure 5:**
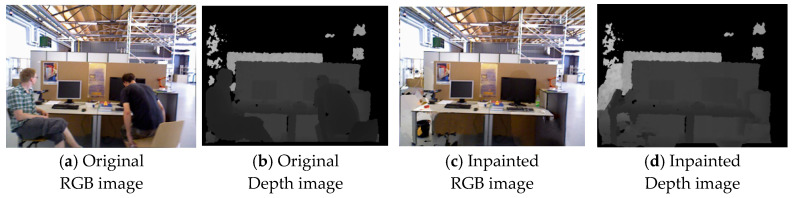
Background inpainting of dynamic objects on the TUM RGB-D dataset.

**Figure 6:**
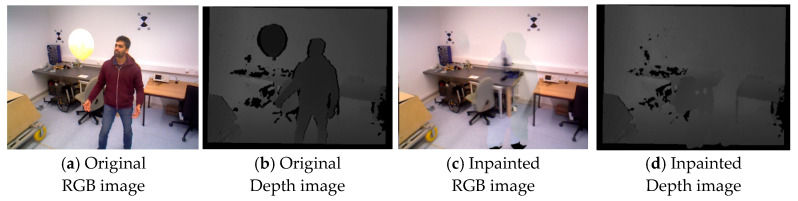
Background inpainting of dynamic objects on the Bonn dataset.

3D Gaussian Splatting requires complete RGB and depth images as input. Therefore, after mask processing, background inpainting is needed for the removed dynamic objects to synthesize a real image without dynamic content. To repair the current segmented frame, it is first aligned with several previous frames that contain visible portions of the target missing area. In the candidate frames, the most suitable source is selected for each pixel to ensure color consistency in the final image [33]. Figure 5 and Figure 6 present the qualitative experimental results, in which (a) and (b) represent the Original RGB image and Original Depth image input into the system, respectively, while (c) and (d) show the system’s output, the Inpainted RGB image and Inpainted Depth image. The experimental results indicate that all dynamic objects in both datasets were successfully detected and segmented, with most of the background effectively inpainted. Only a few small areas showed poor inpainting results, which may be due to those areas not appearing in the previous frames. To achieve more ideal inpainting results, further exploration of more advanced techniques will be necessary in the future.

### 4.3. Registration of 3D Gaussian Splatting

#### 4.3.1. Coarse Registration of 3D Gaussian Submap

(a) Image Matching

For two highly overlapping 3D Gaussian submaps, referred to as the source submap O and the target submap G, each is composed of different keyframes but has not yet been aligned. The goal of this section is to estimate a similarity transformation $T_{O}^{G}$ that aligns O in the coordinate system of G. Due to the arbitrary scale of the 3D Gaussian Submap, the solution for $T_{O}^{G}$ requires considering the scaling issue between O and G. First, the global descriptors of the O and G rendered RGB images are extracted using NetVLAD [34]. NetVLAD is a deep learning-based method that aggregates local features into compact global descriptors by computing residuals between descriptors and learnable cluster centers. In our coarse registration, it extracts global descriptors from rendered 3D Gaussian submaps, enabling robust matching despite scaling, viewpoint changes, or occlusions. Its end-to-end training ensures robustness to environmental variations, while its fixed-dimensional output supports efficient nearest-neighbor search for quick candidate screening.

Then, the cosine similarity between these two sets of images is calculated, and the top 2m pairs are retained for subsequent registration. Image pair can be expressed as:$\left\{\left(I_{O}^{i},I_{G}^{i}\right),i=0,1,\ldots ,2m\right\}$.

(b) Coarse Registration

First, the i-th image match pair $\left(I_{O}^{i},I_{G}^{i}\right)$ is selected, and coarse registration is performed using DUSt3R [35], resulting in a rough transformation matrix $T_{C}^{i}$ (denoted as C for coarse transformation), which includes rotation $R_{C}^{i}$ and translation $t_{C}^{i}$. DUSt3R is a Transformer-based dense 3D registration method whose core lies in estimating dense 3D correspondences and rigid transformations directly from image pairs via an end-to-end network. In the coarse registration stage of this work, DUSt3R serves as a dense geometric correspondence solver, utilizing candidate submap pairs filtered by NetVLAD to directly generate coarse 6-DoF rigid transformation estimates, providing crucial geometric initialization for subsequent fine registration. The adoption of DUSt3R is primarily motivated by three key aspects: first, its insensitivity to initial poses enables it to handle inputs with arbitrary relative poses; second, its attention mechanism models global structural consistency, allowing reliable correspondences to be established even in partially overlapping scenarios; third, the uncertainty measures it outputs assist in screening high-quality transformation estimates.

**Figure 7:**
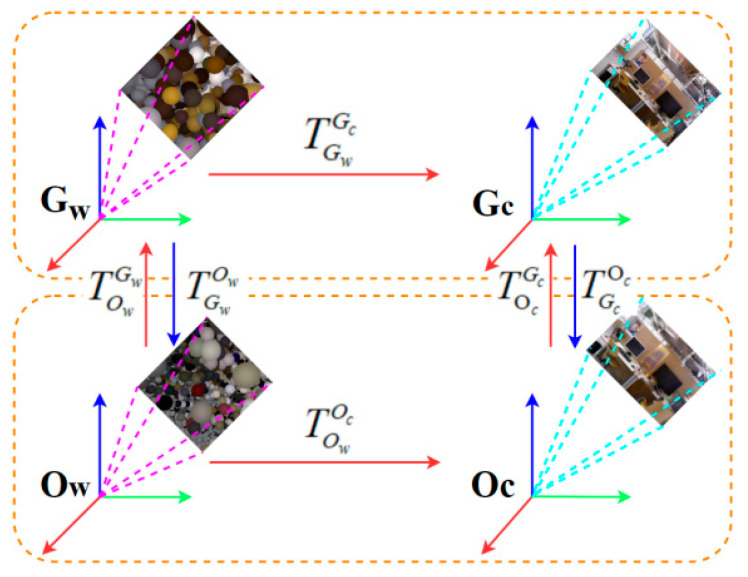
Shows the transformation relationship between different coordinate systems. The subscript w represents the expression of each submap in its respective world coordinate system, while the subscript c represents the expression of each submap’s rendered RGB image in its respective camera coordinate system. The transformation matrix T represents the coordinate system transformation from the subscript to the superscript.

However, due to the unknown scaling factor between O and G, and since DUSt3R takes 2D images as input, the scaling ratio cannot be directly recovered. The obtained transformation matrix $T_{C}^{i}$ needs to be transferred to the two original 3D Gaussian Submaps O and G through coordinate transformation. The transformation relationship between different coordinate systems is shown in Figure 7. The goal is to obtain an accurate rigid transformation $T_{O_{w}}^{G_{w}}$ (where w represents the world coordinate system), which can transform $O_{w}$ to the $G_{w}$ coordinate system, as expressed in Equation (12):

(12) $$G_{w}=T_{O_{w}}^{G_{w}}\cdot O_{w}$$

Based on the coordinate transformation relationship in Figure 7, Equation (12) can be transformed into Equation (13) as shown below:

(13) $$G_{w}=({\left(T_{G_{w}}^{G_{c}}\right)}^{-1}\cdot T_{O_{c}}^{G_{c}}\cdot T_{O_{w}}^{O_{c}})\cdot O_{w}$$

where $T_{O_{w}}^{O_{c}}$ and $T_{G_{w}}^{G_{c}}$ (with c representing the camera coordinate system) respectively represent the transformations of the two submaps O and G from the world coordinate system to the camera coordinate system, corresponding to images $I_{O}^{i}$ and $I_{G}^{i}$. These transformations can be obtained through the tracking module. $T_{O_{c}}^{G_{c}}$ represents the transformation between the two cameras, which includes rotation $R_{O_{c}}^{G_{c}}$, translation $t_{O_{c}}^{G_{c}}$, and scaling $s_{O_{c}}^{G_{c}}$. Here, the rotation and translation of $T_{O_{c}}^{G_{c}}$ match those of $T_{C}^{i}$, so only $s_{O_{c}}^{G_{c}}$ is unknown.

(c) Scale Estimation

Using depth maps corresponding to the camera poses in different coordinate systems, scale estimation $s_{O_{c}}^{G_{c}}$ can be performed. Based on the given camera poses, the depth maps $D_{O}^{i}$ and $D_{G}^{i}$ corresponding to images $I_{O}^{i}$ and $I_{G}^{i}$ are extracted from the 3D Gaussian Submaps O and G, respectively.

In addition, during the coarse registration process using DUSt3R, the depth maps ${\hat{D}}_{O}^{i}$ and ${\hat{D}}_{G}^{i}$ corresponding to images $I_{O}^{i}$ and $I_{G}^{i}$ can also be obtained, along with the corresponding pixel-level confidence maps $C_{O}^{i}$ and $C_{G}^{i}$ for the depth maps. By combining the above depth maps and confidence maps, the distance differences between 3D Gaussian points in the scene can be accurately compared. Here, the depth maps ${\hat{D}}_{O}^{i}$ and ${\hat{D}}_{G}^{i}$ expressed in the same coordinate system and have the same scale. Therefore, the confidence-weighted scale $s_{O_{c}}^{G_{c}}$ between the 3D Gaussian Submaps O and G can be estimated using Equation (14):

(14) $$s_{O_{c}}^{G_{c}}=\frac{C_{G}^{i}⊗\left(D_{G}^{i}/{\hat{D}}_{G}^{i}\right)}{C_{O}^{i}⊗\left(D_{O}^{i}/{\hat{D}}_{O}^{i}\right)}$$

where ⊗ denotes element-wise multiplication. Using $s_{O_{c}}^{G_{c}}$, a preliminary $T_{O_{c}}^{G_{c}}$ can be constructed, which is then used to obtain an $T_{O_{w}}^{G_{w}}$ that allows O to be roughly aligned in the coordinate system of G.

#### 4.3.2. Fine Registration of 3D Gaussian Submap

(a) Photometric Rendering

After O and G are roughly aligned, two sets of images are rendered under a new camera pose $\bar{C}$. The photometric loss function is used to calculate the distance $L_{1}$ between the rendered images [36], with the calculation formula given as Equation (15):

(15) $$L_{1}=l1_{masked}\left(F\left(O,\bar{C}\right),F\left(G,\bar{C}\right),\left(M_{1}⊗M_{2}\right)\right)$$

where F is the differentiable rendering function that generates an image given camera pose and the 3DGS model; $l1_{masked}$ gives the $L_{1}$ distance masked by an element-wise binary mask; and the element-wise binary mask used is $M_{1}⊗M_{2}$, where $M_{1}$ and $M_{2}$ are binary masks indicating whether anything has been rendered at each pixel. We differentiate $L_{1}$ with respect to the parameters of $T_{O_{c}}^{G_{c}}$, given as $R_{O_{c}}^{G_{c}}$, $t_{O_{c}}^{G_{c}}$, $s_{O_{c}}^{G_{c}}$. After the optimization is completed, the rendering residual ε will be saved. Gradient-based optimizers are used to minimize the loss and achieve detailed alignment. In the fine registration stage, photometric registration optimizes pixel-level alignment accuracy between submaps through differentiable rendering technology. Its primary roles include achieving sub-pixel level pose refinement, providing stronger constraints than purely geometric methods in texture-rich regions, and avoiding interference from non-overlapping areas through masking mechanisms. The motivation for its use lies in its high compatibility with differentiable rendering frameworks, as it can leverage image intensity information for optimization without the need for explicit feature matching, and its sensitivity to subtle pose variations, which effectively enhances registration accuracy. This method complements the coarse registration process, together constructing a complete “coarse-to-fine” registration workflow.

(b) Multi-view Pose Optimization

Further optimization of the two sets ${\left\{{\left(T_{O_{c}}^{G_{c}}\right)}_{i}\right\}}_{i=1}^{m}$ and ${\left\{{\left(T_{G_{c}}^{O_{c}}\right)}_{i}\right\}}_{i=m+1}^{2m}$ is performed through multi-view pose optimization to obtain a globally consistent ${\bar{T}}_{O_{c}}^{G_{c}}$, where the first m optimizations come from $T_{O_{c}}^{G_{c}}$, and the last m optimizations come from $T_{G_{c}}^{O_{c}}$. The reciprocal of the above photometric rendering residual ε is used as the weight for each estimate, and weighted rotation averaging is applied to estimate the global rotation [37]. The calculation formula is given as Equation (16):

(16) $${\bar{R}}_{O_{c}}^{G_{c}}=\arg\underset{R_{O_{c}}^{G_{c}}\in SO(3)}{\min}\left({\sum_{i=1}^{m}\frac{1}{\varepsilon_{i}}\left\|R_{O_{c}}^{G_{c}}-{\left(R_{O_{c}}^{G_{c}}\right)}_{i}\right\|}_{F}^{2}+{\sum_{i=m+1}^{2m}\frac{1}{\varepsilon_{i}}\left\|R_{O_{c}}^{G_{c}}-{\left(R_{G_{c}}^{O_{c}}\right)}_{i}^{-1}\right\|}_{F}^{2}\right)$$

where $\|\;{\|}_{F}^{2}$ denotes the Frobenius norm. The global translation ${\bar{t}}_{O_{c}}^{G_{c}}$ and ${\bar{s}}_{O_{c}}^{G_{c}}$ is found as the weighted mean over individual estimates. The final result is a globally consistent ${\bar{T}}_{O_{w}}^{G_{w}}$. Weighted rotation averaging plays a critical role in the multi-view pose optimization stage. It utilizes the reciprocal of photometric rendering residuals as weights to integrate rotation estimates from different submaps, thereby eliminating local inconsistencies caused by single-view errors and achieving globally consistent rotation estimates. The motivation for adopting this method lies in the varying reliability of rotation estimates from different submaps due to disparities in viewpoint, occlusion, or rendering quality. The weighting strategy prioritizes the adoption of more reliable estimation results.

### 4.4. Loop Closure with 3DGS

To effectively detect whether the visual sensor has revisited previously traversed locations, the system incorporates a loop closure detection module [26], which aims to maintain the global consistency of the 3D scene by performing global corrections to the poses of the established submaps and keyframes.

#### 4.4.1. Submap Initialization

The system constructs submaps starting from the first keyframe, with each submap containing a sequence of keyframes observing a specific local region. As the explored scene expands, to avoid the computational burden of maintaining the entire global map at once, the system dynamically triggers the initialization of new submaps. The triggering mechanism is based on the relative motion between the current frame and the first keyframe of the submap: a new submap is created when the translational distance exceeds an empirical threshold $d_{thre}=0.5\;m$ or the rotational angle exceeds an empirical threshold $\theta_{thre}=50{}^{\circ}$. This dynamic threshold mechanism ensures comprehensive scene coverage while effectively controlling the scale of submaps and computational complexity.

#### 4.4.2. Loop Detection and Global Optimization

After a submap is created, the loop detection module is activated. The module first extracts a global descriptor $d\in R^{1024}$ for each keyframe using a pre-trained NetVLAD model, then computes the self-similarity $s_{self}^{i}$ among keyframes within the same submap and the cross-similarity $s_{cross}^{i,j}$ between different submaps. If the cross-similarity between submap i and submap j satisfies $s_{cross}^{i,j}>\min\left(s_{\mathrm{self}}^{i},s_{\mathrm{self}}^{j}\right)$, it is determined that a loop candidate exists. To further suppress false positives, the system additionally calculates the geometric overlap rate between the two submaps, retaining only reliable loops with an overlap rate above an empirical threshold $r_{thre}=0.2$. Upon detecting a valid loop, the system reconstructs the global pose graph, constructs loop edge constraints using the coarse-to-fine 3D Gaussian Splatting registration algorithm proposed in this paper, and ultimately achieves globally consistent registration of all submaps and keyframes through pose graph optimization. This significantly enhances the overall consistency and accuracy of the 3D scene reconstruction.

We evaluated the performance of the loop detection module on the TUM RGB-D and Bonn datasets. The module achieved an average precision of 94.2% and a recall of 88.7% on the TUM RGB-D dataset, and 92.1% precision with 85.3% recall on the Bonn dataset. False positives primarily occur in visually repetitive scenes, while false negatives are associated with extreme viewpoint changes.

## 5. Experiments

### 5.1. Datasets and Evaluation Metrics

This section introduces the experimental setup and compares the proposed system with state-of-the-art baseline methods. The tracking and rendering performance is evaluated on two publicly available real-world dynamic scene datasets—TUM RGB-D [38] and Bonn [17]—and the 3D scene reconstruction results are presented.

The TUM RGB-D dataset, released by the Technical University of Munich in 2012, consists of synchronized RGB-D data collected in indoor scenes using a Kinect sensor, accompanied by high-precision motion capture ground truth. It is a widely used benchmark dataset in the field of dynamic SLAM. The specific sequences from this dataset (e.g., f3/w_h, f3/w_r, f3/w_s, f3/w_x, f3/s_h, f3/s_s, f3/s_x) are selected primarily because they combine human motion (walking and sitting) with multiple camera motion patterns (linear xyz motion, rotational rpy motion, half-sphere arc motion) and static camera scenarios. This effectively simulates real-world scenarios with intertwined static and dynamic objects, providing rich and structured test conditions for evaluating the system’s robustness under dynamic interference. The Bonn dataset, released by the University of Bonn in 2019, collects RGB image data of indoor dynamic scenes using visual sensors and is an important benchmark dataset for autonomous driving and indoor/outdoor dynamic SLAM research. Its sequences (e.g., ball, ball2, ball_tk, mov_no_b, mv_no_b2, mv_o_b2, ps_tk, ps_tk2) are selected mainly because they contain complex dynamic interactions (multi-object motions of pedestrians, balloons, and boxes), effectively validating the algorithm’s robustness and reconstruction accuracy in real indoor dynamic environments. In addition, dedicated ablation studies are conducted to verify the contribution of each proposed module to the system. Finally, the real-time performance of the system is compared and analyzed with the latest 3D Gaussian baselines. All experiments are performed on the same desktop computer with the following configuration: NVIDIA GeForce RTX 3090 GPU (24 GB VRAM; NVIDIA Corporation, Santa Clara, CA, USA), Intel Core i9-11900K CPU (Intel Corporation, Santa Clara, CA, USA), and 32 GB RAM (Samsung, Seoul, Republic of Korea).

### 5.2. Tracking Evaluation

The camera tracking performance is reported in Table 1 and Table 2. Table 1 presents the experimental results on 8 dynamic datasets from TUM RGB-D. Analysis of the experimental data indicates that, among classical SLAM methods, LCD-Splat outperforms baseline approaches such as ORB-SLAM3, ReFusion, LC-SLAM, and YOLO-ORB-SLAM3 on multiple datasets. On the f3/s_x dataset, LCD-Splat is 18.75% and 23.53% lower than DynaSLAM and Yolo-ORB-SLAM3, respectively, and is only 8.33% higher than LC-SLAM. On the average (Avg) of the 8 datasets, LCD-Splat is 84.38%, 60.32%, 10.71%, and 3.85% lower than ORB-SLAM3, ReFusion, LC-SLAM, and Yolo-ORB-SLAM3, respectively. In comparison with the SLAM method of Neural Implicit Fields, the experimental results show that, on some datasets, LCD-Splat performs comparably to the best GO-SLAM, while on other datasets, it outperforms GO-SLAM. On the Avg of the 8 datasets, LCD-Splat is 97.53%, 96.08%, 95.03%, 21.88%, and 54.55% lower than iMAP, NICE-SLAM, ESLAM, GO-SLAM, and RoDyn-SLAM, respectively. In comparison with the 3D Gaussian Splatting method, the experimental results show that LCD-Splat performs similarly to the best DROID-VO on some datasets, while outperforming DROID-VO on others. On the Avg of the 8 datasets, LCD-Splat is 95.04%, 87.86%, 95.44%, 94.88%, and 26.47% lower than SplatTAM, GS-SLAM, LoopSpla, GS-ICP-SLAM, and DROID-VO, respectively.

Table 2 presents the experimental results on 8 dynamic datasets from Bonn. Experimental results show that LCD-Splat outperforms most other baseline methods among classical SLAM approaches. On the ball_tk dataset, LCD-Splat is 5.26%, 88.08%, and 18.18% lower than DynaSLAM, ReFusion, and Yolo-ORB-SLAM3, respectively, and is 12.5% higher than ORB-SLAM3. On the Avg of the 8 datasets, LCD-Splat is 84.37%, 79.51%, and 24.68% lower than ORB-SLAM3, ReFusion, and LC-SLAM, respectively. In comparison with the SLAM method of Neural Implicit Fields, the experimental results show that LCD-Splat performs comparably to the best GO-SLAM on some datasets. On the Avgof the 8 datasets, LCD-Splat is 83.89%, 86.82%, 81.53%, 61.07%, and 52.85% lower than iMAP, NICE-SLAM, ESLAM, GO-SLAM, and RoDyn-SLAM, respectively. In comparison with the 3D Gaussian Splatting method, the experimental results show that, on some datasets, LCD-Splat performs comparably to the best DROID-VO. On the Avg of the 8 datasets, LCD-Splat is 89.08%, 82.42%, 91.57%, 84.53%, and 62.34% lower than SplatTAM, GS-SLAM, LoopSplat, GS-ICP-SLAM, and DROID-VO, respectively.

**Figure 8:**
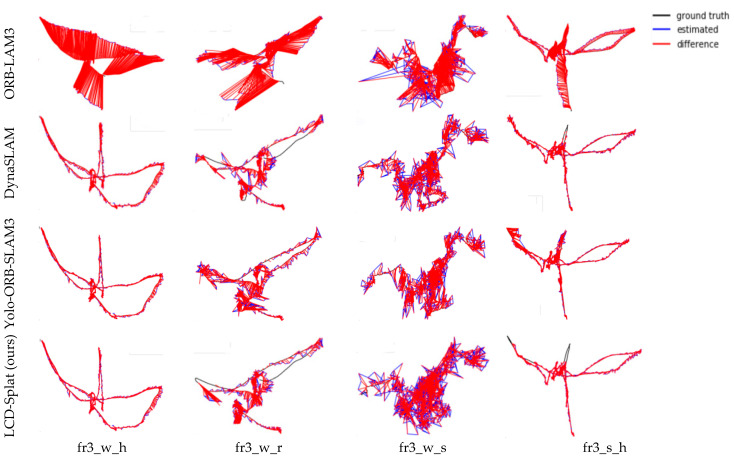
The camera trajectory results estimated by the Advanced Benchmark on the TUM dataset sequences, and the differences with ground-truth values. The black line represents the ground truth trajectory, the blue line represents the estimated trajectory, and the red line represents the difference between the ground truth and the estimated values.

Figure 8 shows the Absolute Trajectory Error (ATE) between the estimated trajectories and the ground truth estimated trajectories and the ground truth trajectories for the ORB-SLAM3, DynaSLAM, Yolo-ORB-SLAM3, and LCD-Splat algorithms on the TUM RGB-D dataset. The differences between the estimated trajectory (in blue) and the ground truth trajectory (in black) are highlighted in red. From observation, it is evident that in highly dynamic sequences such as fr3_w_h, fr3_w_r, fr3_w_s, and fr3_s_h, the original ORB-SLAM3 exhibits significant errors, while the proposed method performs comparably to the advanced benchmark DynaSLAM and Yolo-ORB-SLAM3, generating estimated trajectories that closely match the ground truth.

**Figure 9:**
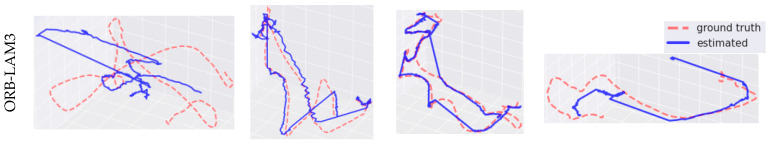

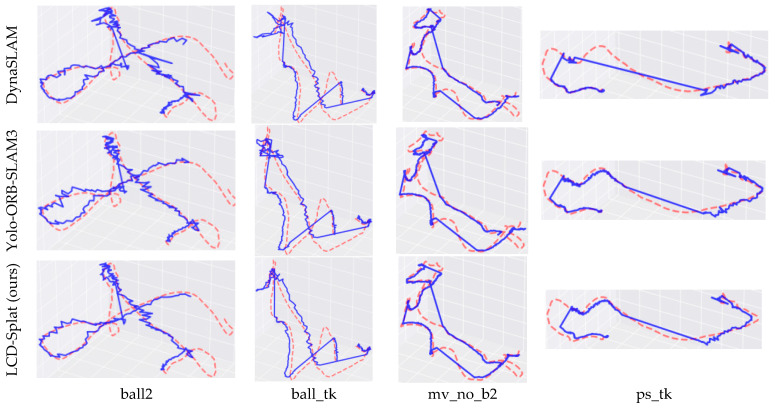
The camera trajectory results estimated by the Advanced Benchmark on the Bonn dataset sequences, along with the differences from the ground truth values, are shown. The ground truth trajectory is represented by the orange line, while the estimated trajectory is represented by the blue line.

Figure 9 illustrates the Absolute Trajectory Error (ATE) between the estimated trajectories and the ground truth trajectories for the ORB-SLAM3, DynaSLAM, Yolo-ORB-SLAM3, and LCD-Splat algorithms on the Bonn dataset. The estimated trajectories are represented by blue lines, while the ground truth trajectories are shown in orange. Upon observation, it is evident that the original ORB-SLAM3 exhibits significant errors in highly dynamic sequences such as ball2, ball_tk, mv_no_b2, and ps_tk. In contrast, the proposed method outperforms Yolo-ORB-SLAM3 and is comparable to the advanced benchmark DynaSLAM, generating estimated trajectories that closely align with the ground truth trajectories.

### 5.3. 3D Scene Reconstruction

**Figure 10:**
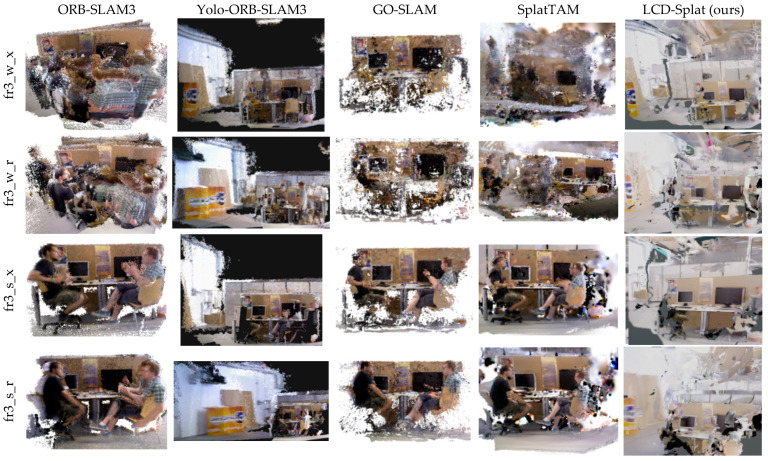
The 3D scene dense reconstruction results of multiple dynamic scene sequences from the TUM RGB-D dataset are shown. LCD-Splat performs excellently in geometric accuracy, robust tracking, and high-quality reconstruction.

**Figure 11:**
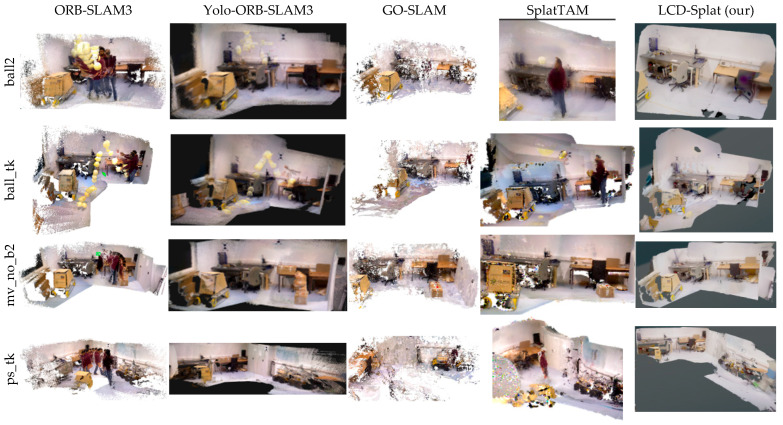
The 3D scene dense reconstruction results of multiple dynamic scene sequences from the Bonn dataset are shown. LCD-Splat performs excellently in geometric accuracy, robust tracking, and high-quality reconstruction.

This paper presents 3D scene dense reconstruction experiments conducted on the TUM RGB-D and Bonn datasets, with the resulting images shown in Figure 10 and Figure 11, respectively. From the reconstruction results, it is evident that the LCD-Splat method outperforms all advanced benchmark methods. In both dynamic datasets, the ORB-SLAM3 algorithm produces floating artifacts in the 3D reconstructed scene due to the presence of moving individuals. The Yolo-ORB-SLAM3 algorithm fails to completely remove the moving individuals from the scene, resulting in a certain degree of artifacts that affect the accuracy of localization and mapping. The GO-SLAM algorithm consumes a large amount of GPU memory during operation, and the presence of moving individuals causes floating artifacts, along with missing local regions in the 3D reconstructed scene. In the 3D scene reconstruction process, SplatTAM experiences suboptimal mapping performance due to the influence of moving individuals, resulting in floating artifacts and incomplete mapping of local regions. In contrast, the LCD-Splat method proposed in this paper demonstrates excellent performance in geometric accuracy, robust tracking, and high-quality reconstruction. This is attributed to the integration of Mask R-CNN and the improved multi-view geometry dynamic object detection module based on 3D Gaussian Splat, as well as the introduction of a novel coarse-to-fine 3D Gaussian registration method, followed by the optimization of global consistency for the 3D Gaussian point cloud.

### 5.4. Rendering Evaluation

This paper conducted a rendering performance analysis on the TUM RGB-D and Bonn datasets, with experimental results shown in Table 3 and Table 4. In the TUM RGB-D dataset experiment, except for the f3/s_s and f3/s_x datasets, where the SplatTAM algorithm has a certain advantage, the proposed LCD-Splat algorithm outperforms other advanced 3D Gaussian algorithms in PSNR, SSIM, and LPIPS metrics in the remaining datasets. In the Bonn dataset experiment, although the LCD-Splat algorithm performs weaker than SplatTAM in the LPIPS metric for certain datasets, it outperforms the current advanced 3D Gaussian Splat benchmarks in other metrics, demonstrating significant advantages.

**Figure 12:**
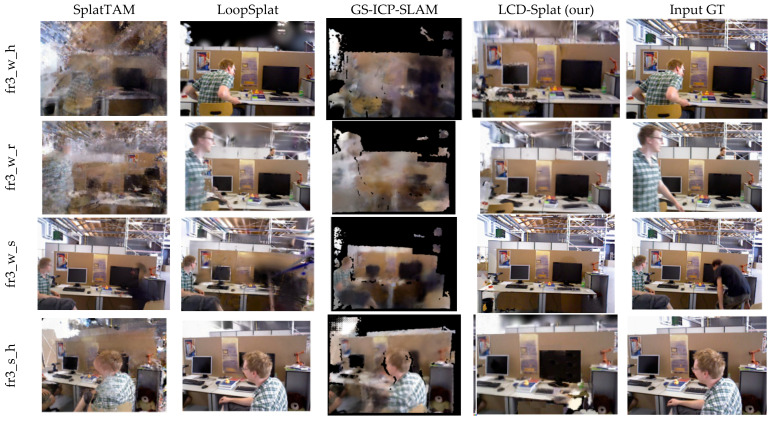
It shows the visual comparison of rendering images on the TUM RGB-D dataset. The results presented in this paper are more complete and accurate, unaffected by the floating artifacts caused by dynamic objects.

**Figure 13:**
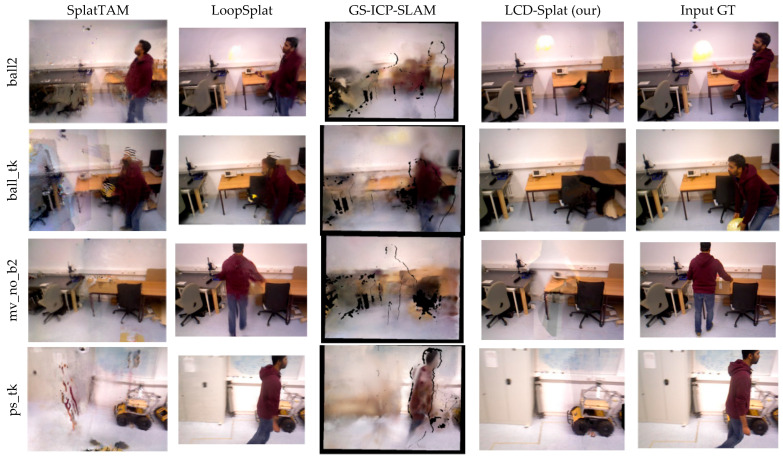
It shows the visual comparison of rendering images on the Bonn dataset. The results presented in this paper are more complete and accurate, unaffected by the floating artifacts caused by dynamic objects.

Figure 12 and Figure 13 respectively display the rendering results of the LCD-Splat method and other advanced 3D Gaussian Splat methods on the TUM RGB-D and Bonn datasets. The benchmark methods suffer from varying degrees of blurring and shadows due to the interference of dynamic objects, with incomplete rendering in local regions and an inability to produce high-fidelity static maps. In contrast, using the LCD-Splat method proposed in this paper for static map reconstruction achieves high-fidelity rendering. This indirectly demonstrates that, compared to other mainstream SLAM systems, the method presented in this paper can generate more accurate static maps in highly dynamic scenes.

### 5.5. Ablation Study

To evaluate the effectiveness of the proposed system, and considering that the TUM RGB-D dataset’s semantic priors cover major motion categories, such as moving individuals, while the Bonn dataset contains some undefined dynamic objects, such as balloons and boxes, an ablation study is conducted on 8 representative sequences from the Bonn dataset to avoid interference from prior information. The average value of the ATE (m) metric is used to quantify the impact of each component on the overall system performance. The ablation study results are shown in Table 5.

Through comparison with multiple baseline methods, the effectiveness of the proposed approach in enhancing camera tracking performance has been validated. Specifically, after incorporating the Mask R-CNN and improved multi-view geometry dynamic object detection module, the coarse-to-fine 3D Gaussian registration method, and the 3D Gaussian based loop closure detection module, the overall system performance has been significantly improved. As shown in Table 5, the introduction of the loop closure detection module reduced the absolute trajectory error (ATE) from 0.191 m to 0.058 m, representing an improvement in tracking accuracy of approximately 69.6%.

### 5.6. Time Consumption Analysis

To comprehensively evaluate the real-time performance of the proposed system, we conducted a comparative experiment on the per-frame average total runtime using mainstream SLAM systems, including NICE-SLAM, ESLAM, Point-Slam, SplaTAM, and the proposed LCD-Splat system, under dynamic scenarios based on the TUM RGB-D dataset. The results are shown in Table 6. As indicated in the table, the average total runtime of the proposed system (LCD-Splat) is 3.167 s, which is significantly lower than that of SplaTAM (8.414 s), slightly better than ESLAM (3.799 s) and Point-Slam (3.916 s), and superior to NICE-SLAM (4.887 s). It is noteworthy that under similar runtime conditions, the proposed system outperforms comparable methods such as LoopSplat in both camera trajectory accuracy and map reconstruction quality, demonstrating a superior trade-off between efficiency and accuracy. This experimental result further validates the effectiveness of the lightweight tracking module and multi-level geometric verification mechanism designed in this paper, providing a more practical solution for real-time 3D reconstruction in dynamic scenes.

To thoroughly investigate the impact of each core module on the overall system runtime and to clarify the distribution of computational load across different processing stages, this study conducted a timing analysis of the processing pipeline of LCD-Splat on the TUM RGB-D dataset. As shown in Table 7, the processing time for the loop closure detection module is 0.264 s, accounting for approximately 8.34% of the total per-frame processing time of 3.167 s. This indicates that loop closure detection effectively enhances the global consistency of the system while maintaining reasonable computational overhead, without significantly impacting overall real-time performance. In terms of the time distribution among modules, the Mask R-CNN-based dynamic object removal module incurs the highest computational cost, with a processing time of 0.549 s, primarily due to the high computational complexity of pixel-level instance segmentation algorithms. The 3D Gaussian registration module follows closely, consuming 0.357 s, which reflects the computational load associated with iterative Gaussian parameter optimization and dense scene representation. The improved multi-view geometry-based completion module requires 0.328 s and is mainly used to identify potentially moving objects that are easily overlooked. Overall, the system achieves a reasonable allocation of computational resources among key tasks while maintaining robustness in dynamic environments and high-fidelity reconstruction accuracy.

### 5.7. Deployability Analysis and Efficiency Optimization Strategies

To enhance the practical deployment capability of the proposed system on resource-constrained platforms such as mobile devices and embedded robots, this paper explores a systematic optimization strategy. By applying structured pruning to core modules such as the depth estimation network and the dynamic segmentation model, computational complexity and memory usage can be significantly reduced while algorithmic accuracy is maintained. Building on this, an adaptive inference mechanism can be further designed to flexibly adjust the execution frequency of each module according to dynamic scene characteristics. For instance, in static scenarios, the processing frequency of computationally intensive modules could be reduced, whereas the tracking module would maintain high-frequency operation, thereby achieving dynamic optimization of computational load. Leveraging the characteristics of edge hardware, inference engines such as TensorRT can be employed for graph optimization and operator fusion, enabling efficient deployment on low-power platforms like the Jetson series. It should be noted that the above lightweighting and deployment strategies have not yet been fully experimentally validated, and their practical effectiveness and specific deployment performance still require further verification in future work. This integrated optimization scheme provides a potential technical pathway for the practical application of dynamic SLAM systems in resource-constrained environments.

## 6. Conclusions

This paper proposes a 3D Gaussian SLAM algorithm specifically designed for dynamic environments. The algorithm integrates Mask R-CNN and an improved multi-view geometry-based dynamic object detection module, which removes dynamic features from the scene while preserving static regions as much as possible. It eliminates dynamic areas and feature points at mask edges that may affect matching accuracy, while retaining feature points in static regions. Additionally, the paper introduces a coarse-to-fine 3D Gaussian registration method and optimizes the global consistency of the 3D Gaussian point cloud, thereby enhancing the reliability of the SLAM system in dynamic environments. However, the current method still has some limitations. First, LCD-Splat, when processing dynamic environments, especially during fine segmentation using Mask R-CNN, operates at a slower speed and requires higher-performance GPU support, making it challenging to achieve real-time performance for the entire system to enable practical applications. Second, the reliability of the submap registration-based scale estimation method proposed in this paper still requires further validation and improvement in challenging scenarios such as low-texture, highly dynamic, or extreme lighting conditions. Future work will focus on the following directions: primarily, designing a more lightweight dynamic object segmentation module to enhance the real-time performance of the LCD-Splat algorithm; and simultaneously exploring multi-sensor fusion and adaptive depth completion mechanisms to strengthen the stability and generalization capability of submap registration-based scale estimation in complex environments.

## Figures and Tables

**Table 1:** Camera tracking performance (ATE RMSE ↓ [m]) on multiple dynamic scene sequences in the TUM RGB-D dataset. * indicates the results reproduced from the official code of this paper, X denotes tracking failure, and — means not mentioned. For the top three results in each dataset, different colors are used for highlighting.

Method	f3/w_h	f3/w_r	f3/w_s	f3/w_x	f3/s_h	f3/s_r	f3/s_s	f3/s_x	Avg
Classical									
* ORB-SLAM3 [14]	0.434	0.198	0.012	0.493	0.038	0.080	0.010	0.013	0.160
* DynaSLAM [15]	0.029	0.031	0.007	0.016	0.021	0.043	0.007	0.016	0.021
EM-fusion [16]	5.100	—	1.400	6.600	3.200	—	0.900	3.700	3.483
* ReFusion [17]	0.104	X	0.017	0.099	0.110	X	0.009	0.040	0.063
MID-fusion [18]	0.038	—	0.023	0.068	0.031	—	0.010	0.062	0.039
LC-SLAM [19]	0.041	0.055	0.016	0.024	0.022	—	—	0.012	0.028
* Yolo-ORB-SLAM3 [20]	0.029	0.041	0.008	0.016	0.037	0.053	0.007	0.017	0.026
Neural Implicit Fields									
iMAP [9]	—	1.395	1.373	1.115	0.930	—	—	0.236	1.010
* NICE-SLAM [10]	X	X	0.882	1.138	0.450	X	X	0.079	0.637
* ESLAM [21]	0.608	0.904	0.936	0.457	0.036	—	—	0.076	0.503
* GO-SLAM [22]	0.025	0.099	0.024	0.014	0.021	0.018	0.033	0.018	0.032
RoDyn-SLAM [23]	0.056	0.078	0.017	0.083	0.044	—	—	0.051	0.055
3D Gaussian Splatting									
* SplatTAM [13]	1.077	0.727	0.610	1.342	0.144	0.109	0.004	0.017	0.504
GS-SLAM [25]	X	0.335	0.084	0.377	X	X	X	0.027	0.206
* LoopSplat [26]	1.012	1.528	0.199	1.377	0.064	0.145	0.036	0.019	0.548
* GS-ICP-SLAM [27]	0.665	1.234	0.934	0.752	0.129	0.074	0.031	0.086	0.488
DROID-VO [39]	—	0.100	0.007	0.017	—	—	—	0.011	0.034
* LCD-Splat (our)	0.033	0.036	0.009	0.017	0.025	0.055	0.011	0.013	0.025

**Table 2:** Camera tracking performance (ATE RMSE ↓ [m]) on multiple dynamic scene sequences in the Bonn dataset. * indicates the results reproduced from the official code of this paper, X denotes tracking failure, and — means not mentioned. For the top three results in each dataset, different colors are used for highlighting.

Method	ball	ball2	ball_tk	mov_no_b	mv_no_b2	mv_o_b2	ps_tk	ps_tk2	Avg
Classical									
* ORB-SLAM3 [14]	0.209	0.178	0.032	0.134	0.036	0.861	0.719	0.799	0.371
* DynaSLAM [15]	0.028	0.027	0.038	0.032	0.030	0.020	0.054	0.088	0.040
* ReFusion [17]	0.175	0.254	0.302	0.071	0.179	0.528	0.289	0.463	0.283
LC-SLAM [19]	0.033	0.026	—	0.021	—	0.295	0.044	0.045	0.077
* Yolo-ORB-SLAM3 [20]	0.036	0.030	0.044	0.039	0.043	0.267	0.051	0.041	0.069
Neural Implicit Fields									
iMAP [9]	0.149	0.670	0.248	—	0.283	—	0.283	0.528	0.360
* NICE-SLAM [10]	X	0.668	0.212	X	0.319	X	0.549	0.453	0.440
* ESLAM [21]	0.226	0.362	0.124	X	0.177	X	0.480	0.514	0.314
* GO-SLAM [22]	0.040	0.027	0.032	0.023	0.028	0.172	0.317	0.551	0.149
RoDyn-SLAM [23]	0.079	0.115	0.133	—	0.126	—	0.145	0.138	0.123
3D Gaussian Splatting									
* SplatTAM [13]	0.360	0.341	0.131	0.151	0.187	0.595	1.204	1.279	0.531
GS-SLAM [25]	0.375	0.268	0.319	X	0.048	X	0.468	0.504	0.330
* LoopSplat [26]	0.628	0.332	0.423	0.549	0.464	0.878	1.173	1.059	0.688
* GS-ICP-SLAM [27]	0.209	0.367	0.455	0.220	0.362	0.266	0.644	0.473	0.375
DROID-VO [39]	0.054	0.046	0.089	—	0.059	—	0.214	0.460	0.154
* LCD-Splat (our)	0.052	0.031	0.036	0.037	0.034	0.125	0.053	0.095	0.058

**Table 3:** The rendering performance on the TUM RGB-D dataset is presented. For each dataset, the best-performing metric is highlighted in bold black for emphasis.

Method	Metrics	f3/w_h	f3/w_r	f3/w_s	f3/w_x	f3/s_h	f3/s_r	f3/s_s	f3/s_x	Avg
SplatTAM	PSNR	16.03	15.28	19.88	17.39	18.31	16.31	23.68	**22.24**	18.64
	SSIM	0.602	0.568	0.816	0.662	0.755	0.582	**0.898**	**0.873**	0.720
	LPIPS	0.392	0.41	0.187	0.332	**0.240**	0.37	**0.118**	**0.173**	0.278
GS-ICP-SLAM	PSNR	16.18	16.54	18.14	16.96	18.47	17.79	18.15	18.03	17.53
	SSIM	0.683	0.703	0.722	0.708	0.734	0.719	0.685	0.719	0.709
	LPIPS	0.409	0.384	0.332	0.374	0.303	0.348	0.344	0.363	0.357
LoopSplat	PSNR	14.99	15.96	X	15.85	18.67	19.24	X	20.61	17.55
	SSIM	0.596	0.564	X	0.524	0.419	0.408	X	0.234	0.458
	LPIPS	0.473	0.531	X	0.563	0.694	0.725	X	0.852	0.640
LCD-Splat (ours)	PSNR	**22.05**	**20.47**	**30.24**	**20.52**	**21.56**	**21.17**	**30.07**	21.92	**23.50**
	SSIM	**0.842**	**0.944**	**0.854**	**0.839**	**0.813**	**0.796**	0.861	0.683	**0.829**
	LPIPS	**0.343**	**0.235**	**0.135**	**0.313**	0.338	**0.28**	0.145	0.376	**0.271**

**Table 4:** The rendering performance on the BONN dataset is presented. For each dataset, the best-performing metric is highlighted in bold black for emphasis.

Method	Metrics	ball	ball2	ball_tk	mov_no_b	mv_no_b2	mv_o_b2	ps_tk	ps_tk2	Avg
SplatTAM	PSNR	18.93	16.39	18.67	20.76	20.43	17.12	16.39	14.92	17.95
	SSIM	0.73	0.694	0.796	0.851	0.813	0.719	0.646	0.555	0.726
	LPIPS	**0.255**	**0.302**	**0.233**	**0.193**	**0.21**	0.299	0.303	0.382	0.272
GS-ICP-SLAM	PSNR	14.92	15.45	17.43	17.27	17.37	17.89	15.19	14.96	16.31
	SSIM	0.72	0.701	0.743	0.741	0.754	0.756	0.716	0.699	0.729
	LPIPS	0.345	0.363	0.351	0.355	0.33	0.315	0.360	0.375	0.349
LoopSplat	PSNR	22.33	19.27	23.99	21.55	22.02	24.18	21.11	21.1	22.06
	SSIM	0.825	0.717	0.836	0.791	0.791	0.861	0.765	0.763	0.794
	LPIPS	0.339	0.438	0.356	0.373	0.395	0.307	0.413	0.387	0.376
LCD-Splat (our)	PSNR	**23.7**	**21.26**	**27.4**	**29.26**	**29.07**	**26.53**	**27.28**	**24.94**	**26.18**
	SSIM	**0.829**	**0.772**	**0.848**	**0.881**	**0.869**	**0.863**	**0.865**	**0.842**	**0.837**
	LPIPS	0.311	0.313	0.259	0.233	0.255	**0.242**	**0.256**	**0.269**	**0.267**

**Table 5:** Ablation study on Bonn datasets. “Imp MVG” refers to the improved multi-view geometry method, while “Reg 3D GS” refers to the coarse-to-fine 3D Gaussian registration method.

LCD-Splat (ours)	Mask R-CNN	Imp MVG	Reg 3D GS	Loop Closure	ATE (m)
	√	X	X	√	0.385
	X	X	√	√	0.396
	√	√	X	X	0.191
	√	X	√	√	0.087
	√	√	√	√	0.058
Baseline	SplatTAM				0.531
	GS-SLAM				0.330
	LoopSplat				0.688
	GS-ICP-SLAM				0.375
	DROID-VO				0.154

**Table 6:** Run-time comparison on TUM RGB-D datasets.

Time Module	NICE-SLAM	ESLAM	Point-Slam	SplaTAM	LoopSplat	LCD-Splat (ours)
Avg. Total Running (s)	4.887	3.799	3.916	8.414	3.007	3.167

**Table 7:** Computational Time Breakdown Analysis for LCD-Splat System key components.

Module Name	Time Consumption (s)
Mask R-CNN	0.549
Improved Multi-view Geometry	0.328
Reg 3D GS	0.357
Loop Closure	0.264

## Data Availability

Data will be made available on request.
